# Miracle Fruit, a Potential Taste-modifier to Improve Food Preferences: A Review

**DOI:** 10.1007/s13668-024-00583-3

**Published:** 2024-10-03

**Authors:** Shashya Diyapaththugama, Getahun Fentaw Mulaw, Madiha Ajaz, Natalie Colson Shilton, Indu Singh, Rati Jani

**Affiliations:** 1 School of Pharmacy and Medical Sciences, Griffith University, Gold Coast, QLD 4222 Australia; 2 School of Health Sciences and Social Work, Griffith Health, Griffith University, Gold Coast, QLD 4222 Australia

**Keywords:** *Synsepalum*, *Synsepalum dulcificum*, Miracle berry, Taste perception, Taste modification, Sweetener

## Abstract

**Purpose of Review:**

The miracle fruit contains the glycoprotein miraculin which can modify the taste perception of food and beverages at low pH conditions, altering the consumers’ food preferences. This review aims to critically evaluate all available evidence on miracle fruit/ miraculin and taste modification and its potential role in improving food preferences.

**Recent Findings:**

Miracle fruit suppresses sourness and induces sweetness in acidic food/ beverages. At low pH conditions, miracle fruit enhances the sweet taste and decreases the perceived intensities of salty and bitter tastes in solutions. However, the role of miracle fruit in sweet, salty, and bitter food is not adequately studied. The above effects alter the food-liking scores in individual foods and mixed diets.

**Summary:**

Miracle fruit is a pH-dependent taste modifier with the potential to be used in food applications to improve consumer food preferences. Future research on the changes in food preferences with the optimum miraculin dose, food type, and intrapersonal variations in taste sensitivity is warranted.

**Supplementary Information:**

The online version contains supplementary material available at 10.1007/s13668-024-00583-3.

## Introduction

Miracle fruit or *Synsepalum dulcificum* (Schumach. & Thonn. Daniell), previously known as *Richadella dulcifica,* is an evergreen shrub belonging to the Sapotaceae family, and native to West Africa. The red berry grown in the plant, having an average weight of 1.11 ± 0.17 g, consists of skin, pulp, shell, and seed, from outside to inside [1]. It is highly perishable and can be freeze-dried at -20^0^F for six months [2]. The terms “miracle fruit” and “miracle berry” are used interchangeably in literature to refer to the red berry. This paper consistently uses the term “miracle fruit”. Miracle fruit contains a glycoprotein called “miraculin”, which has a unique taste-modifying property. Miraculin is a homodimer composed of 191 amino acids, nitrogen, carbohydrates, and sugars (glucosamine, mannose, fructose, xylose, and galactose) and has a molecular weight of 24,000 to 45,000 Da [3–5]. Miraculin which is originally tasteless, activates the human sweet taste receptors at pH below 6.5 and converts the perception of sour foods into a sweet taste comparable to sucrose [2, 6, 7]. Miraculin is thermolabile (inactivated above 100 °C) and gets inactivated at pH below 3 and above 12. Miracle fruit pulp which is acidic with a pH of 3.3 ± 0.14 is the only part that contains miraculin [1, 8]. The miraculin content in the pulp varies from 0.07 to 1.30 mg/g of juice [9]. The nutrient and phytochemical profile of the miracle fruit pulp is provided in Online Resource 1.

The unique pH-dependent taste modification property of miraculin has drawn the attention of researchers. A miracle fruit consumed prior to an acidic fruit such as lime or lemon, can disguise the sour taste perception and induce a sweetness [10]. Researchers have attempted to propose various explanations for miraculin’s taste modification mechanism of action. Initially, it was described as a paralysis of some papillae of the tongue, a blocking of sour receptors preventing the perception of sour taste, or the addition of sweetness to sour acids by miraculin [11]. However, other scientists have claimed that the effect of miraculin on sourness reduction is due to changes in the taste receptors at low pH conditions, causing induction of sweetness rather than an action of intrinsic suppression of sourness [7, 12].

**Fig. 1:**
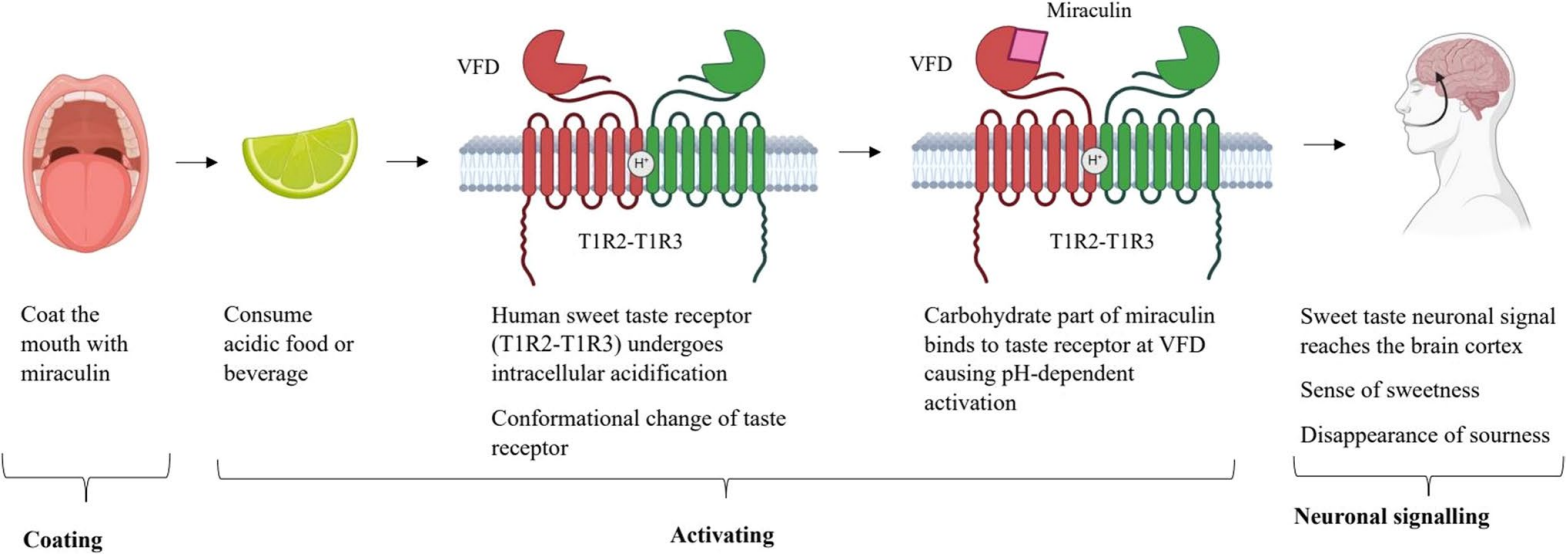
Taste-modification mechanism of action of miraculin. Created with BioRender.com

Human sweet taste receptors are made up of two types of proteins, belonging to the G protein-coupled taste receptor family. They are, taste receptor type 1 membrane 2 (T1R2) (coded by gene *TAS1R2*), and taste receptor type 1 membrane 3 (T1R3) (coded by gene *TAS1R3*). T1R2 responds to small, sweet molecules while T1R3 responds to large ones [12, 13]. The human sweet taste receptor has three main parts: the transmembrane domain, the N-terminal segment Venus Flytrap Domain (VFD), and the cysteine-rich domain [12]. The transmembrane domain is involved in signal transduction across the cell membrane. VFD is responsible for ligand binding. Cysteine-rich domain maintains the structural stability of the receptor [13]. Within the VFD, there are two forms: free form I (resting state) and free form II (resting state). These two forms are in dynamic equilibrium. When a ligand binds to free form II, the receptor is stabilized [2, 10]. Miraculin’s mechanism of action can be discussed in three steps as demonstrated in Fig. 1: coating the mouth, activating, and neuronal signalling.

Coating the mouth: Chewing of miracle fruit results in the taste buds being coated with miraculin [14]. Miraculin interacts with the VFD, form II of the sweet taste receptors [15].

Activating the taste receptor: In the presence of extracellular acidification, the extracellular region of the T1R2 and miraculin gets protonated, leading to the activation. Full receptor activation occurs with intracellular acidification [15]. The epithelial plasma membrane of the sweet taste receptor undergoes a conformational change at acidic pH, e.g. 4.8–6.5. Then, the carbohydrate part of the miraculin molecule binds to the sweet taste receptor. This results in the pH-dependent receptor activation [2, 16, 17]. The polypeptide chain of the miraculin contains histidine residues located in exposed regions which play an important role in the above-mentioned conformational change of the homodimer of miraculin [6].

Neuronal signalling: The taste fibres called S-fibres which usually convey taste from T1R2/ T1R3 receptors responding only to sweet taste, now become responsive to acids too after miraculin administration. When the sweet taste neuronal signal reaches the cortex of the brain, the sour taste disappears [2, 10].

As the presence of H^+^ ions is essential for the above mechanism to occur, miracle fruit consumed alone does not produce a sweet taste. The action may last from 30 s after the consumption of the fruit up to 2 h, producing a sweet taste each time a sour food/drink is tasted until salivary amylase dissociates the miraculin from taste receptors [2, 10, 16]. To elucidate the mechanism of miraculin action in the human brain, magnetic fields of the cerebral cortex were recorded using Magnetoencephalography (MEG) on seven subjects, aged 22–35 years, in Japan. Based on the observations, it was suggested that after chewing miracle fruit, the sourness component from citric acid was diminished to a greater extent and the sweetness information reached the primary taste area of the cerebral cortex [18].

Due to miraculin’s taste modification mechanism, miracle fruit is explored in literature as a taste modifier. The basic taste qualities, sweet, sour, bitter, salty, umami, and fat affect the food liking. Humans have biological biases towards liking sweet, fatty, and salty food while disliking bitter and sour food, with individual variations in taste liking such as bitter taste sensitivity and psychophysiological characteristics [19]. The bitter taste is associated with the isothiocyanates derived from glucosinolates of brassica vegetables. The isothiocyanates demonstrate antioxidant, anti-inflammatory, anti-diabetic, and chemoprotective effects [20]. Due to the health benefits of phytochemicals in vegetables, it is suggested to mask the bitterness to facilitate the consumption of vegetables [21]. The sour taste is associated with organic acids in fruits [19, 22]. Organic acids prevent osteoporosis, inhibit platelet aggregation, produce intestinal hormones, and provide anti-inflammatory, and anti-obesity effects [23]. Thus, miracle fruit can be suggested as a potential pH-dependent strategy to sweeten the food by masking the sour and bitter tastes which may improve fruit and vegetable consumption.

Miracle fruit is researched for two aspects: (i) health applications and (ii) taste modification. While the health applications have been extensively reviewed [3, 12, 24–26], there are no comprehensive reviews on taste modification. Existing reviews on health applications (e.g. antidiabetic, anticancer, antioxidant, hepatoprotective, anti-hyperlipidemic, and anti-hyperuricemia) are summarized in Online Resource 2. These direct health benefits are predominantly provided by the phytochemicals such as flavonoids, tannins, alkaloids saponin, etc. in miracle fruit, rather than its ability to modify taste. While taste modification can indirectly encourage healthy eating habits by making certain foods more palatable, the direct health benefits are attributed to the phytochemicals. Taste modification caused by miraculin in miracle fruit is researched on sour, sweet, bitter, salty tastes, and taste combinations in solutions, selected food, and mixed diets. However, no review had been undertaken to synthesize these taste modification studies. The present review addresses this gap by compiling all the available scientific evidence on taste modification studies conducted with miracle fruit/ miraculin; comprehensively synthesizing and analysing them; and discussing the potential role of incorporating miracle fruit to mask/alter the taste perception to improve preferences/intake of healthy food such as fruits, vegetables, and yoghurt. By examining these scenarios, the review identifies the research methodologies that yield positive results and any limitations.

## Method

### Justification of Methodology

The present review adopted both systematic and narrative review methodologies [27]. The studies on taste modification effects of miracle fruit on sour, bitter, sweet, salty, or mixed taste qualities (Table 1) were predominantly quasi-experimental with quantitative outcomes (Table 2). Therefore, a systematic search strategy was a useful approach to critically evaluate and select relevant original research evidence [27]. A narrative approach was used in synthesis due to the heterogeneity of independent, dependent variables, and outcome measurements, the unavailability of statistical approaches in some studies, to theoretically interpret the reasons for significant and non-significant results, and to narratively outline the future directions [28, 29]. As this was the first review that investigated the results of taste modification studies of miracle fruit, the narrative synthesis was appropriate.

**Table 1:** List of the 16 studies selected for the present review and the taste qualities studied in each study

Study No	Author (year)	Taste quality				
		Sour	Sweet	Bitter	Salty	Combinations of two or more tastes
1	Kurihara and Beidler (1969) [32]	*				
2	Bartoshuk et al. (1974) [11]	*				
3	Capitanio et al. (2011) [7]	*	*	*	*	*
4	Igarashi et al. (2013) [33]	*				
5	Rodrigues et al. (2016) [34]	*				
6	Haddad et al. (2020) [35]	*				
7	Hudson et al. (2018) [36]	*,○	*			
8	Andrade et al. (2019) [37]	*		*		
9	Lipatova and Campolattaro (2016) [38]	*, ○	○	○	○	
10	Wong and Kern (2011) [39]	○				
11	Choi and Garza (2020) [14]	○				
12	Choi and Garza (2021) [40]	○				
13	Endo et al. (2015) [41]	*				
14	Choi and Park (2023) [42]	○			○	○
15	López et al. (2020) [43]	*				
16	Wilken and Satiroff (2012) [44]					○

*Studying the effect of miracle fruit on solutions/beverages, ○studying the effect of miracle fruit on food

**Table 2:** A summary of key characteristics and findings of the included articles

Author (year), Country	Study design	Age, gender, ethnicity	Sample size	Taste	Independent variable/ Treatment	Dependent variable (taste measurement)	Results	Direction of association/ Conclusions
Kurihara and Beidler (1969) [32], Japan	Pre-post study	Not mentioned	6	Sour	Miraculin application before tasting 0.02 M citric acid	Perceived sweetness		Sweetness ↑ (No statistical approach)
Bartoshuk et al. (1974) [11], United States	Pre-post study	Not mentioned	9	Sour	25 mg miracle fruit tablets application before tasting HCl (0.00078 M to 0.01288 M) and citric acid (0.00085 M to 0.0138 M)	Perceived sweetness	Mean ± SEM of sourness ↓, from 100.2 ± 11.7 to 16.3 ± 5.6 in 0.014 M citric acid, from 108.9 ± 25.8 to 45.7 ± 12.2 in 0.013 M HCl	No significant difference (*p* > 0.05) between the post-test sweetness of HCl and citric acid
Bartoshuk et al. (1974) [11], United States	Pre-post study	Not mentioned	10	Sour	Citric acid + sugars (sucrose, L-arabinose, D-xylose) Miracle fruit + citric acid	Perceived sweetness		Sourness ↓ Sweetness ↑. (No statistical approach)
Bartoshuk et al. (1974) [11], United States	Pre-post study	Not mentioned	12	Sour	0.01 M HCl solutions paired with seven acids; acetic, ascorbic, citric, gluconic, malic, sulfuric, and tartaric acids. Each pair consisted of HCl and a test acid giving equal sourness. Miracle fruit administration before each acid	Perceived sweetness	The ratio of sweetness, citric: HCl = 2.43, tartaric: HCl = 1.86, malic:HCl = 1.48, sulfuric: HCl = 1.41, ascorbic: HCl = 1.33, acetic:HCl = 1.23, gluconic:HCl = 1.08	Citric acid and tartaric acid were significantly (*p* < 0.07) sweeter than HCl
Capitanio et al. (2011) [7], Italy	Pre-post study	21.6 ± 2.2 years, 30% male, 70% female,	10	Sour	25mcg of miraculin application before tasting 0.01 M citric acid	Perceived sourness and sweetness on a scale from 0–100	Sourness score: ↓ Sweetness score: ↑	Sourness ↓ Sweetness ↑ significantly (*p* < 0.001)
Igarashi et al. (2013) [33], Japan	Pre-post study	~ 20 years, 44.4% male, 55.5% female	18	Sour	Freeze-dried miracle fruit application before tasting lemon juice, juice of *Citrus depressa*, grain vinegar, apple cider vinegar, and white-wine vinegar	Perceived sourness and sweetness on a scale from 0–200	Mean ± SEM of sweetness: 133.2 ± 8.5 in lemon juice, 119.1 ± 9.2 in *Citrus depressa*, 94.4 ± 6.9 in grain vinegar, 96.2 ± 7.5 in apple cider, and 91.7 ± 9.9 in white-wine vinegar	Lemon juice significantly (*p* < 0.01) sweeter than grain vinegar, apple cider, and white-wine vinegar The juice of *Citrus depressa* significantly (*p* < 0.05) sweeter than grain vinegar and white-wine vinegar
Rodrigues et al. (2016) [34], Brazil	Balanced complete block design	27 ± 4.6 years, 75% female, 25% male	12	Sour	4 groups, (i) Lemonade (unsweetened) (ii) Lemonade + sugar, (iii) Lemonade + sucralose, (iv) Miracle fruit (300 mg) + lemonade	Sweetness intensity over time (TI), dominant sensation using Just-about-right-scale (a linear 9 cm scale)	Maximum sweetness intensity:1.59 ± 1.02 in (i), 6.49 ± 1.76 in (ii), 5.76 ± 1.98 in (iii), and 4.98 ± 2.00 in (iv) Area under the TI curve: 37.45 ± 18.31 in (i), 197.97 ± 79.22 in (ii), 168.85 ± 74.25 in (iii), and 159.95 ± 78.15 in (iv)	Significant (*p* ≤ 0.05) difference in TI parameters (maximum intensity, area under the TI curves) between (i) and (iv) for sweetness. No significant difference (*p* ≤ 0.05) among TI effects of sucralose and miracle fruit Sweet sensation dominant in (iii) and (iv)
Haddad et al. (2020) [35], Lebanon	Complete block design		10	Sour	4 types of lemonades, (i) Lemonade (unsweet) (ii) Lemonade + sugar, (iii) Lemonade + aspartame, (iv) Miracle fruit powder + lemonade	Perceived sourness and sweetness on a line-scale	Highest sourness in (i) (10.53), highest lemon taste in (i) (11.82), highest sweetness in (iv) (11.63)	Miracle fruit ↑ sweetness in lemonade (iv)
Hudson et al. (2018) [36], United States	Pre-post study	18–55 years, 50% male, 50% female	97	Sour	Miracle fruit application before tasting ‘predominantly sour’ (apple cider vinegar, lemons, yellow mustard, pickle chips), ‘both sour and sweet’ (tomatoes, strawberries), and ‘control’ (chicken sausages, unsalted peanuts) food	Perceived sourness and sweetness on Global Sensory Intensity Scale from 0–100	Sour ratings ↓ (vinegar 48–20, lemon 47–13, mustard 26–11, pickle 25–11, strawberry and tomato 10–2) Sweetness ratings ↑ (vinegar 6–32, lemon 5–38, mustard 6–26, pickle 6–19, strawberry 24–45, tomato 10–31)	Significant ↑ (*p* < 0.05) in sweetness and ↓sourness in ‘Predominantly sour’ food and ‘both sour and sweet’ food Sausages (control food) ↑ in sweetness
Andrade et al. (2019) [37], Brazil	Balanced complete block design	20–35 years, 73% female, 27% male	11	Sour	6 groups, (i) Unsweetened lemonade (ii) 150 mg miracle fruit + lemonade (iii) 300 mg miracle fruit + lemonade (iv) 600 mg miracle fruit + lemonade (v) Lemonade + sucrose (vi) Lemonade + sucralose	Sweetness intensity over time (TI), dominant sensation, liking on a 9-point hedonic scale	Maximum sweetness intensity with miracle fruit 150 mg was 5.77, 300 mg was 6.94, 600 mg was 7.3. The area under the TI curve was 217.94 for 150 mg, 236.63 for 300 mg, and 278.28 for 600 mg Liking for unsweetened lemonade was 4.75 ± 1.8, the group with miracle fruit 300 mg was 6.30 ± 1.9, sucrose was 7.05 ± 1.6, sucralose was 6.95 ± 1.4	Similar maximum sweetness intensity for 300 mg and 600 mg (*p* > 0.005) and significantly different (*p* ≤ 0.005) from 150 mg. Highest area under the TI curve for 600 mg (p ≤ 0.005) Similar consumer liking for 300 mg miracle fruit, sucrose, and sucralose and significantly higher (p ≤ 0.005) than unsweetened lemonade
Lipatova and Campolattaro (2016) [38], United States	Pre-post study	Undergraduate students	19	Sour	Chewing of fresh miracle fruit before tasting lemon, grapefruit, lime, sour candy, and apple cider vinegar	Perceived sweetness on a scale from 0–10	Mean ± SEM of sweetness ↑, sourness ↓ in all food items	Sweetness ↑ and sourness ↓ significantly (*p* < 0.01)
Wong and Kern (2011) [39], United States	Randomized cross-over design	Not mentioned	13	Sour	4 desserts, (i) regular lemon-based popsicle having sucrose (REG) (ii) miracle fruit + regular popsicle (REG + MF) (iii) low-sugar popsicle (DIET) (iv) miracle fruit + low sugar popsicle (DIET + MF)	Perceived sweetness on a 100 mm visual analogue scale	Mean ± SD values for sweetness (mm): 72 ± 23 for REG, 70 ± 21 for REG + MF, 29 ± 38 for DIET, and 58 ± 36 for DIET + MF	REG (*P* < 0.01), REG + MF (*P* < 0.01), and DIET + MF (*P* < 0.05) were significantly sweeter compared to DIET
Choi and Garza (2020) [14], United States	Quantitative Descriptive Analysis using a Latin Square design	27.3 ± 3.9 years, 80% female, 20% male, 50% Whites, 30% Hispanics, 20% Asians	10	Sour	Miracle fruit application (600 mg tablet, 300 mg tablet, 400 mg tablet, 300 mg powder) before tasting green apple, goat cheese, lemonade, yogurt, and cucumber pickle	Perceived sweetness on a 15 cm line scale labelled from “weak” to “strong”	The mean difference between pre and post-test sweetness: 400 mg tablet + yoghurt = 8.47, 600 mg + yoghurt = 8.33, 600 mg + cheese = 7.54, 400 mg + cheese = 7.45, 350 mg + yoghurt = 5.36	Sweetness ↑ significantly (*p* < 0.01) in all food with all 4 miracle fruit products. The sweetening effects varied with the miracle fruit product and the type of food
Choi and Garza (2021) [40] United States	Pre-post study	18–65 years, 55% female, 45% male,	200	Sour	Miracle fruit application (600 mg tablet, 300 mg tablet, 400 mg tablet, 300 mg powder) before tasting green apple, goat cheese, lemonade, yogurt, and cucumber pickle	Food preferences on a 9-point hedonic scale from 1 “dislike extremely” to 9 “like extremely”	The mean difference between pre and post-test overall liking was, apple (1.08) > goat cheese > (0.96) > yoghurt (4.26)	Liking scores significantly ↑ (*p* < 0.05) in yogurt, goat cheese, and apple Liking scores ↓ in lemonade and pickle
Endo et al. (2015) [41], Japan	Pre-post study	20.7 ± 0.8 years, 50% male, 50% female	20	Sour (acid mixtures)	350 mg of powdered miracle fruit tablet before tasting single, binary, and trinary mixtures of citric, malic, tartaric, and acetic acids	Perceived sweetness, sourness, and astringency on a 7-point scale from -3 to + 3	Significant differences in sourness and astringency at *p* < 0.05 and *p* < 0.01 between different individual and combinations of solutions	
Choi and Park (2023) [42], United States	Randomized placebo-controlled crossover trial	60.2 ± 8.8 years, 50% male, 50% female, Korean-American, diabetes or prediabetes patients	50	Sour	Miracle fruit tablet or placebo application before tasting green apple, goat cheese, lemonade, yogurt, and cucumber pickle	Food preferences on a 9-point hedonic scale from 1 “extremely dislike” to 9 “extremely like”	Mean differences in pre and post-test liking after miracle fruit: yoghurt 3.08 ± 0.19, cheese 2.48 ± 0.16, apple 2.16 ± 0.13, pickle 1.84 ± 0.17, lemonade 1.76 ± 0.18. After placebo: yoghurt 0.52 ± 0.15, cheese 0.30 ± 0.13, apple 0.38 ± 0.09, pickle 0.60 ± 0.11, lemonade 0.36 ± 0.15	Overall liking significantly ↑ (*p* < 0.001) in the miracle fruit group compared to placebo
López et al. (2020) [43], Mexico	Pre-post, study	(i) Patients with inborn errors in metabolism (IEM)- 43% female, 57% male, 15.8 years (ii) Healthy subjects- 50% male, 50% female, 31.7 years	21 (7 IEM, 14 healthy)	Sour	Miracle fruit application before tasting vinegar, lemon, and acidic liquid metabolic formula	Pre- and post-acceptance on a 5-point hedonic scale from 1 (hate it) to 5 (loved it)	4/7 patients and 8/14 healthy subjects had positive changes in perception of the metabolic formula	Significant ↑ in liking for metabolic formula (p = 0.021) in healthy subjects
Capitanio et al. (2011) [7], Italy	Pre-post study	21.6 ± 2.2 years, 30% male, 70% female	10	Sweet	25mcg of miraculin application before tasting, (i) 0.037 M sucrose (ii) 0.037 M sucrose + 0.01 M citric acid	Perceived sweetness on a scale from 0–100	Pre and post-test mean sweetness scores ↑ in (ii)	Sweetness ↑ significantly (*p* < 0.001) in (ii), the binary solution of sucrose + citric acid
Lipatova and Campolattaro (2016) [38], United States	Pre-post study	Undergraduate students	19	Sweet	Chewing of fresh miracle fruit before tasting jellybeans	Perceived sweetness on a scale from 0–10	Pre and post-test mean sweetness scores were 8.5 and 8.5	No changes (*p* > 0.01) (due to absence of low pH condition)
Hudson et al. (2018) [36], United States	Pre-post study	18–55 years, 50% male, 50% female	97	Sweet	Miracle fruit application before tasting, ‘predominantly sweet’ food (dark chocolate, maple syrup)	Perceived sweetness on Global Sensory Intensity Scale from 0–100	Sweetness ratings in pre and post-test changed from, 38 to 40 in maple syrup, 32 to 36 in chocolate	No changes (*p* > 0.05) (due to absence of low pH condition)
Capitanio et al. (2011) [7], Italy	Pre-post study	21.6 ± 2.2 years, 30% male, 70% female,	10	Bitter	25mcg of miraculin application before tasting, (i) 0.005 M caffeine (ii) 0.005 M caffeine + 0.01 M citric acid	Perceived bitterness on a scale from 0–100	Pre and post-test scores ↓ in (ii)	Bitterness ↓ significantly (*p* < 0.001) in (ii), the binary solution of caffeine + citric acid
Andrade et al. (2019) [37], Brazil	Balanced complete block design	20–35 years, 73% female, 27% male	11	Bitter	6 groups, (i) Unsweetened green tea (ii) 150 mg miracle fruit + green tea (iii) 300 mg miracle fruit + green tea (iv) 600 mg miracle fruit + green tea (v) green tea + sucrose (vi) green tea + sucralose	Bitterness intensity over time (TI), dominant sensation, liking on a 9-point hedonic scale	Maximum bitterness intensity with miracle fruit 150 mg = 4.51, 300 mg = 4.76, 600 mg = 4.34, sucrose = 4.18. Similar area under the TI curve for all miracle fruit groups. Highest sweetness intensity was 4.76 in sucrose Liking for unsweetened green tea = 5.29 ± 2.13, miracle fruit 300 mg = 5.36 ± 1.91, 600 mg = 5.30 ± 1.79, sucrose = 5.71 ± 2.14	No significant difference (*p* > 0.05) between the maximum bitterness intensities of 150 mg, 300 mg, 600 mg miracle fruit groups, and sucrose Sucrose provided significantly higher sweetness intensity (*p* < 0.05) and highest liking (*p* ≤ 0.005)
Lipatova and Campolattaro (2016) [38], United States	Pre-post study	Undergraduate students	19	Bitter	Chewing of fresh miracle fruit before tasting raw broccoli	Perceived bitterness on a scale from 0–10	Pre and post-test mean bitterness scores were 4.5 and 4.5	No changes (*p* > 0.01) (due to absence of low pH condition)
Capitanio et al. (2011) [7], Italy	Pre-post study	21.6 ± 2.2 years, 30% male, 70% female,	10	Salty	25mcg of miraculin application before tasting, (i) 0.034 M NaCl (ii) 0.034 M NaCl + 0.01 M citric acid	Perceived saltiness on a scale from 0–100	Pre and post-test scores ↓ in (ii)	Saltiness ↓ significantly (*p* < 0.001) in (ii), the binary solution of NaCl + citric acid
Lipatova and Campolattaro (2016) [38], United States	Pre-post study	Undergraduate students	19	Salty	Chewing fresh miracle fruit before tasting salty crackers	Perceived saltiness on a scale from 0–10	Pre and post-test mean saltiness scores were 6.5 and 5	No changes (*p* > 0.01) (due to absence of low pH condition)
Capitanio et al. (2011) [7], Italy	Pre-post study	21.6 ± 2.2 years, 30% male, 70% female,	10	Mixed solutions	25mcg of miraculin application before, (i) citric acid + caffeine + sucrose (ii) citric acid + NaCl + caffeine (iii) citric acid + NaCl + sucrose	Perceived taste on a scale from 0–100	Post-test, sourness in (i) ↓, Sweetness in (i), (ii), and (iii) ↑ Saltiness in (ii) and (iii) ↓	Sourness in (i)↓ (*p* < 0.005) Saltiness ↓ in (ii) (*p* < 0.005) and (iii) (*p* < 0.0001)
Wilken and Satiroff (2012) [44], United States	Placebo-controlled, cross-over, pilot trial	47–69 years, 88% female, 12% male, chemotherapy patients	8	Mixed diets	Miracle fruit tablet or placebo application before consuming meals	Perceived taste and food intake recorded on a food diary. (ticked as better/same/worse), and comments	All participants reported positive changes with miracle fruit compared to placebo	Metallic taste, sourness ↓, sweetness ↑, improved food intake. (Sample size small to report statistical significance)
Choi and Park (2023) [42], United States	Randomized placebo-controlled crossover trial	60.2 ± 8.8 years, 50% male, 50% female, Korean-American, diabetes or prediabetes patients	50	Mixed diets	Miracle fruit tablet or placebo application before consuming breakfast and dinner meals	Food preferences on a 9-point hedonic scale from 1 “extremely dislike” to 9 “extremely like”. Calorie intake by weighing food and using an online nutrient tracker (https://cronometer.com/)	Miracle fruit group: liking for, breakfast 7.3 ± 0.7, lunch 7.4 ± 0.5, dinner 7.9 ± 0.4. Placebo group: liking for, breakfast 6.0 ± 0.9, lunch 6.2 ± 0.6, dinner 6.6 ± 0.7. Calorie intake from miracle fruit to placebo groups: breakfast 1377 ± 185 to 1521 ± 202, lunch 2329 ± 688 to 2470 ± 691, dinner: 5203 ± 487 to 5360 ± 495	Overall liking for breakfast and dinner ↑ significantly (*p* < 0.05). Significantly ↓ (*p* < 0.001) calorie intake in the miracle fruit group compared to placebo

## Data Sources and Search Strategies

The literature search was performed between June 2023 to September 2023 in the electronic scientific databases MEDLINE (Ovid), CINAHL, Scopus, and Web of Science. The search was conducted using the Boolean operators (AND/OR) and appropriate truncations. The Population, Intervention, Comparison, Outcome (PICO) framework was used in defining the research question in which humans (P), receiving miracle fruit/ miraculin (I), were compared with the pre-test or placebo group (C), on the outcomes of perceived taste quality, taste intensity, food preferences/liking, dietary intake, and diet quality (O). The PICO framework and keyword search strategy are summarized in Online Resource 3. Keywords used for the exposure variable included miracle fruit, miracle berry, *Synsepalum dulcificum*, *Richadella*, and miraculin. Keywords used for the outcome variable included taste, taste perception, taste modification, taste sensitivity, food preference, food liking, diet, and diet quality.

Reference lists of the selected articles were also checked for potentially qualifying research. The PRISMA flowchart [30] is provided in Online Resource 4. Sixteen articles with original research work were included in the present review.

## Selection Criteria

Only publications in English were included. Publications from all years were considered as the research in this area was limited. Animal studies, in-vitro studies, unpublished reports, conference abstracts, letters to the editors, editorials, and commentaries were excluded. Two researchers (SD and GM) independently reviewed the abstracts and full texts using Covidence software (https://www.covidence.org/). Any disagreements were addressed by a third reviewer (RJ). Cohen's kappa (κ) inter-rater agreement was calculated. The values are interpreted as follows: < 0.00 as no agreement, 0.00–0.20 as slight, 0.21–0.40 as fair, 0.41–0.60 as moderate, 0.61–0.80 as substantial, and 0.81–1.00 as perfect agreement [31]. The κ inter-rater agreement (κ = 0.911, 95% CI: 0.789–1.000) was within the perfect agreement category.

## Results and Discussion

### Study Characteristics

In total, 16 studies conducted to determine the effect of miracle fruit/ miraculin on taste perception and food preferences in human participants were included in the review. Study designs are reported in Online Resource 5. Table 1 provides a list of the selected 16 studies and the taste qualities focused by each study. Table 2 summarizes the key characteristics and findings of the selected 16 articles. More than half (*n* = 13/16) of the studies were conducted after 2011, mostly in Caucasian populations from Western nations (e.g., United States, Brazil, Italy, Lebanon), involving healthy adults (*n* = 14/16), diabetes/pre-diabetes (*n* = 1/16), chemotherapy patients (*n* = 1/16), and patients with inborn errors of metabolism (*n* = 1/16). Sample sizes ranged from 6 to 200 including participants of age ranging from 18–65 years and both genders. Perceived taste intensities were measured on scales ranging from 0–100 (*n* = 2), 0–200 (*n* = 1), 0–10 (*n* = 1), and other line scales such as Just-about-right-scale (9 cm scale) (*n* = 1), 100 mm visual analogue scale (*n* = 1), and 15 cm line scale (*n* = 1). Food preferences were measured on 9-point hedonic scales (ranging from 1-extremely disliked to 9-extremely liked) in *n* = 3 studies and 5-point hedonic scales in *n* = 1 studies. The effect of miracle fruit was experimented in sour (*n* = 15/16), sweet (*n* = 3/16), bitter (*n* = 3/16), salty (*n* = 2/16) tastes, and combinations of two or more tastes (*n* = 3/16).

## Sour Taste

Table 2 reports that the majority of available studies (*n* = 15/16) examined the effect of miracle fruit on sour taste using sour acids (*n* = 5/15), juices/beverages (*n* = 4/15), and food (*n* = 6/15). Miracle fruit effects were commonly tested with citric acid as the sour acid (*n* = 4/5), lemon juice/ lemonade as sour beverages (*n* = 4/4), and lemon and lime as food items (*n* = 6/6). Miracle fruit significantly decreased the sourness, increased the sweetness, or improved the food liking in *n* = 13/15 studies. The findings agree with the mechanism of action (Fig. 1) that, miracle fruit produces a sweet taste in the presence of acidic pH [2, 10, 16].

While acidity is essential for the taste modification action, the effectiveness also depends on the specific pH of the acid. In vitro study by Sanematsu et al. (2016) [17] investigated the relationship between receptor activation and pH within the range of 4.8 to 7.4. When the pH became more acidic (decreasing from 6.5 to 4.8), the taste receptor cells showed a stronger response to miraculin. This stronger response is attributed to the increased protonation of the taste receptor at lower pH levels, which enhances the binding affinity and receptor activation. Conversely, at neutral pH levels (6.5 to 7.4), protonation is minimal, resulting in a very little response as the receptor remains in a less active or inactive conformation [17].

Although Bartoshuk et al. (1974) [11] observed no significant difference between the sweetness in HCl and citric acid, the authors suggested that perceived sweetness intensity varies with the type of the acid. In agreement with the above suggestion, Igarashi et al. (2013) [33] observed that the acidity created by citric acid-containing solutions is more effective for miraculin activity compared to that of acetic acid-containing solutions.

The literature which investigated the effect of miracle fruit on sour food has also agreed with the above finding as Hudson et al. (2018) [36] observed that not all sour foods were equally sweetened by miracle fruit. Weak acids produce a more undissociated form which enters the intracellular spaces of receptor cell membrane inducing intracellular acidification. Intracellular pH is associated more closely with miraculin activity. Therefore, weak acids are more effective than strong acids in producing taste-modifying effects with miraculin [15]. The possibility of utilizing miracle fruit as a substitute for sugars and other sweeteners in commercial food production was investigated subsequently in lemonade by Rodrigues et al. (2016) [34] and in lemon-based popsicles by Wong and Kern (2011) [39]. Based on the findings the authors recognized miracle fruit as a potential sugar substitute in lemonade and lemon juice-based popsicles [34, 39]. However, pilot tests have suggested that miracle fruit should not be included as an ingredient in food but should only be coated on the tongue before the consumption of food to generate sweetening effects [25, 39]. This can be attributed to the mechanism of action of miraculin where a time gap of 30 s is needed for the miraculin to act on acidified taste receptors while the maximum sweetening effect will be given at 3 min [32]. Furthermore, as miraculin is a glycoprotein, it is deactivated by heat making it unsuitable to be cooked as a food ingredient [40].

## Sweet Taste

According to Table 2, *n* = 3 studies were conducted on sweet taste using sucrose solutions (*n* = 1/3) and sweet food (*n* = 2/3). At neutral pH, miraculin did not alter the perceived sweetness intensity of 0.037 M sucrose solution, jellybeans, dark chocolate, and maple syrup [7, 36, 38]. However,, miraculin increased the perceived sweetness of the binary mixture containing sucrose and citric acid [7]. Agreeing with the mechanism of action, the findings confirm the requirement of an acidic pH condition for miraculin’s activity [7, 15]. The authors further proposed that the elevated sweetness of sucrose solutions is caused by the additive effect of the sweet taste of sucrose and the sweet taste induced by miraculin’s action on human sweet taste receptors [7].

According to the in-vitro cell-assay based observations by Koizumi et al. (2011) [17], when a sweet-tasting substance was administered at a neutral pH to the receptor cells pre-incubated with miraculin, the activity of the newly administered sweet-tasting substance was inhibited. The authors indicate that the interaction between miraculin and sweet taste receptors is very strong and miraculin prevents further receptor activation by a second sweet-tasting compound [16, 17]. Accordingly, conflicting results were obtained on sweet taste at neutral pH. The human studies reported no change in the sweetness of food [37, 39] while the in vitro study reported a decrease in sweetness [17].

## Salty Taste

Table 2 reports, *n* = 2 studies conducted on salty taste using sodium chloride (NaCl) (*n* = 1/2) and salty crackers (*n* = 1/2), which claim the requirement of a low pH condition to decrease the perceived salty taste intensity. Like sweet taste, miraculin did not alter the perceived saltiness in solutions or food [7, 38]. However, Capitanio et al. (2011) [7] observed a significant decrease in salty taste in a binary solution containing NaCl and citric acid. Although the authors considered the above results to be ambiguous, their explanation for the mechanism of saltiness suppression by miraculin was that the sweetness elicited by miraculin lowered the salt taste perception, under the acidic environment inside the mouth. The availability of only *n* = 2 studies could also lead to conflicting results due to interpersonal variation.

## Bitter Taste

According to Table 2, *n* = 3 studies were conducted with miraculin/ miracle fruit on bitter taste. No significant changes in bitterness were observed in caffeine, green tea, or broccoli [7, 37, 38]. However, Capitanio et al. (2011) [7] observed a significant decrease in bitterness of the binary mixture containing caffeine and citric acid. Based on the results, it can be suggested that miraculin may decrease the perceived bitterness in the presence of low pH conditions. The limited number of studies (*n* = 3) may lead to conflicting results caused by interpersonal variation.

## Combination of Tastes

Table 2 reports that the effect of miraculin on combinations of more than two tastes had been experimented on *n* = 3 studies using solutions on healthy adults (*n* = 1/3) and mixed diets on diabetes/pre-diabetes and chemotherapy patients (*n* = 2/3). Positive changes such as the increase in overall food liking and improvement in food intake were observed with mixed diets while the effect of miraculin on solutions provided variable results [7, 42, 44].

Enhancement of sweetness and suppression of sourness at different degrees was observed in solutions containing combinations of two or more tastes. Solutions representing sour, sweet, salty, and bitter tastes were mixed to simulate the actual mixed diet eating conditions. A significant reduction in sourness was observed only in the citric acid + caffeine + sucrose mixture. The reduction in sourness was non-significant in citric acid + NaCl + caffeine mixture and in citric acid + NaCl + sucrose mixtures. Although the decrease in sourness was inconsistent, a significant sweetness was produced in each mixture, irrespective of the presence of sucrose [7].

According to Capitanio et al. (2011) [7], the observations of miraculin on bitter taste were inconsistent in solutions. Although miraculin suppressed the bitterness in a binary mixture of citric acid and caffeine, there was no significant decrease in bitterness in solutions containing caffeine + citric acid + sucrose or caffeine + citric acid + NaCl (Table 2). It remains questionable why the miraculin did not act as a pH-dependent sweetener in this instance, in a similar mechanism as with salty taste. One possible reason may be the higher variability of the effects of sweetness on bitter taste while the suppressive effects of sweetness on salty taste were more stable [7].

## Role of Miracle Fruit on Food Preference and Intake

The effects of miracle fruit on food preferences were observed in *n* = 3 studies conducted with selected individual sour food/beverages (*n* = 3/3) and mixed diets (*n* = 1/3) claiming the possibility of miracle fruit to alter food liking [37, 40, 42]. Andrade et al. (2019) [37] observed that miracle fruit increased the liking of unsweetened lemonade According to Choi and Garza (2021) [40], miracle fruit enhanced the overall liking of yoghurt, goat cheese, and apple while decreasing the liking of lemonade and cucumber pickle in healthy adults. However, in diabetic/pre-diabetic adults miracle fruit has improved the liking towards yoghurt, goat cheese, apple, lemonade, and cucumber pickle compared to a placebo [42]. In terms of food intake, miracle fruit facilitated the food intake in chemotherapy patients and helped the diabetes patients to replace sugar, reducing calorie intake [42, 44]. Accordingly, taste alterations resulting from miracle fruit can either enhance or suppress the liking/ intake of food depending on the dose of miraculin, type of food, gender, and intrapersonal differences in sensitivity to taste and aroma.

Furthermore, discrepancies were observed in the miracle fruit-induced food liking across studies. In healthy adults, Choi (2021) [39] observed a decrease in liking for lemonade, while Andrade [37] observed an increase in liking for lemonade. The liking for lemonade was increased in diabetes/pre-diabetes patients too [37]. These variations across studies may be attributed to the differences in taste sensitivity of participants groups.

## Toxicity and Side Effects of Miracle Fruit

A number of miracle fruit product brands are available in the market in the United States and no adverse effects caused by consumption of the products were reported by the US FDA Adverse Event Reporting System (FAERS) or the Centre for Food Safety and Applied Nutrition (CFSAN) Adverse Event Reporting System (CAERS) [45]. Toxicity and allergenicity experiments were conducted in vivo and in silico to assess the safety of miracle fruit for consumption [24, 45].

Animals were administered with varied doses of miracle fruit or seed extracts to study the toxicity. The acute toxicity study conducted by Chinelo and Uzoma (2015) [46] demonstrated that oral administration of miracle fruit pulp extract, up to a dose of 5000 mg/kg was not toxic enough to cause death of the albino rats. However, *Synsepalum dulcificum* seed extract caused death of mice at doses of 5, 10, 20, 100, and 1000 mg/kg, while the dose of 2.5 mg/kg did not cause death. Furthermore, LD_50_ (the dose that causes the death of 50% of animals), which is an indicator of acute toxicity, for the *Synsepalum dulcificum* seed extract was calculated as 3.54 mg/kg [47]. Therefore, Ee et al. (2022) [24] suggest conducting further studies to evaluate the toxicity of miracle fruit in humans.

Potential allergenicity and toxigenicity of miraculin in humans were investigated by in silico bioinformatic analysis. According to the findings, miraculin did not demonstrate allergenicity or toxigenicity risks to humans [45]. Further in silico experiments reported the absence of cross-allergy between miracle fruit and peanut concluding that miracle fruit is safe for individuals with peanut allergy [48]. Furthermore, sensory evaluation of miracle fruit powder administration to six participants indicated that the taste-modifying effects commenced and disappeared rapidly and did not cause a desensitization effect [45].

## Future Directions

Future directions are discussed on two key areas: requirement for future research and practical applications in food industry and healthcare. Further research should be conducted to determine the effect of miracle fruit on the taste perception of food items. Based on the available evidence in Table 2, miraculin in miracle fruit can decrease the perceived sourness in acidic food [14, 36, 39, 40]. However, the effect of miracle fruit on food items representing sweet, bitter, and salty tastes was not adequately studied. The available studies that observed the change in taste perception of sweet, bitter, and salty food items did not supply a low pH condition which is essential for miraculin’s mechanism of action [36, 38]. Therefore, in future pre-post quasi-experimental studies should be conducted to determine the change in perceived taste intensities of food items representing sweet, bitter, or salty tastes after the administration of miracle fruit and an acid such as lime juice.

It can be suggested to conduct systematic dose–response studies with miracle fruit. According to Table 2, Choi and Garza (2020) [14] and Andrade et al. (2019) [37] conducted dose–response studies on miracle fruit and taste perception of sour and bitter tastes. Choi and Garza (2020) [14] conducted a pre-post taste perception study in the United States with different doses and forms of miracle fruit products (e.g. 300 mg tablet, 400 mg tablet, 300 mg powder) obtained from different manufacturers. The authors identified that the post-test sweetness scores vary with the form and dose of miracle fruit products. However, the findings do not provide an adequate comparison among the doses of miracle fruit due to the differences in ingredients, composition, and form (e.g. powder, tablet). The balanced complete block design study conducted by Andrade et al. (2019) [37] in Brazil compared the effects of three doses of miracle fruit tablets (150 mg, 300 mg, 600 mg) obtained from the same manufacturer on taste perceptions of lemonade (sour) and green tea (bitter). However, the authors have not supplied a low pH condition (acids) with the bitter green tea samples which is essential to activate miraculin [15]. Furthermore, there are no available studies on the change in perception of sweet and salty tastes with different doses of miracle fruit. Therefore, future studies should be conducted with a selected miracle fruit product (e.g. fresh fruit, freeze-dried fruit, tablet, or powder) available in the market to observe whether the change in dose can increase or decrease the perceived intensities of individual tastes. For this, multi-arm trials such as randomized controlled trials or quasi-experimental studies can be proposed [49]. Furthermore, during the data collection, a low pH condition should essentially be maintained in the mouth to activate miraculin [15].

The role of miracle fruit on dietary management should be researched further. According to Table 2, miracle fruit improves food consumption in diabetes/pre-diabetes patients [42] and chemotherapy patients [44]. No other diseased populations were studied with miracle fruit. Future long or short-term interventions can be conducted in participants with other chronic disease conditions such as obesity and cardiovascular diseases to determine the efficacy of miracle fruit in improving food preferences and diet quality.

The effect of miracle fruit on diets of healthy adults should be studied in future. Furthermore, the effect of miracle fruit to overcome picky eating behaviours in children can be explored. According to Table 2, there are no available studies on the effect of miracle fruit on the food preferences of mixed diets among healthy adults or children. Future studies should explore the potential of miracle fruit to improve the preferences for nutritious food and diet quality in healthy populations as an approach to prevent the chronic diseases [50, 51]. For this, long-term interventional studies can be suggested.

According to available evidence, miracle fruit acts as a sugar substitute in lemonade and popsicles [34, 37]. Coating the mouth with miracle fruit may play a potential role in the food product development in future food industry as a sugar substitute in confectionary items, mask sour/bitter tastes of fruits and vegetables, and create novel flavour profiles [34, 37]. Furthermore, miracle fruit may play a role in the dietary management of obesity, diabetes, and cancer in future [42, 44]

## Conclusions

The taste modification property of miraculin in miracle fruit was studied on individual or combinations of tastes using solutions, beverages, and food. Miracle fruit can decrease the perceived sourness and increase the sweetness in acidic food/beverages. Miracle fruit alters the perception of sweet, bitter, and salty tastes, only at low pH conditions. The alterations in food liking and preferences caused by miraculin/ miracle fruit depend on the dose of miraculin, the type of tested food, and intrapersonal variations in taste sensitivity. Miracle fruit is a pH-dependent taste modifier with the potential to improve food preferences.

## Key References

- Choi S, Garza J. Consumer Likings of Different Miracle Fruit Products on Different Sour Foods. Foods 2021, 10, 406. 2021.
  This pre-post quasi-experimental study identifies the factors that affect the action of miracle fruit. The study concludes that the effect of miracle fruit changes with the form of miracle fruit product, the manufacturer, dose, and the type of food.

- Choi SE, Park T. Feasibility and acceptability of miracle fruit application prior to the consumption of sour-tasting foods as a weight-loss strategy in adults with diabetes or prediabetes: A randomized crossover trial. Appetite. 2023:107046.
  This randomized placebo-controlled trial investigated the role of miracle fruit on calorie intake and food liking in humans. This study investigated the effect of miracle fruit on mixed diets using an adequate sample size. According to the findings, miracle fruit may be a solution to control non-communicable diseases such as diabetes.

- López L, Vela M, Ibarra I, Díaz L, Belmont L, Guillén S. Positive improvement in palatability of metabolic formula with the use of miraculin protein in patients with inborn errors of metabolism and healthy adults. Rev Chil Nutr. 2020;47(5):801–7.
  This pre-post quasi-experimental study compares the effect of miracle fruit on sour liquids and a metabolic formula. This study compared two population groups: healthy participants vs patients with inborn errors of metabolism. This is the first study that suggested to use miracle fruit to improve the palatability of metabolic formulas for patients.

## Supplementary Information

Below is the link to the electronic supplementary material.

Supplementary file1 (DOCX 24 KB)

Supplementary file2 (DOCX 23 KB)

Supplementary file3 (DOCX 16 KB)

Supplementary file4 (DOCX 53 KB)

Supplementary file5 (DOCX 28 KB)

## Data Availability

No datasets were generated or analysed during the current study.

## Abbreviations

T1R2: Taste receptor type 1 membrane 2
T1R3: Taste receptor type 1 membrane 3
VFD: Venus Flytrap Domain
US FDA: United States Food and Drug Administration
