# Supplementary material for: Miracle Fruit, a Potential Taste-modifier to Improve Food Preferences: A Review
Source: Curr Nutr Rep. 2024 Oct 3;13(4):867–83. doi: 10.1007/s13668-024-00583-3 (PMC11489218; doi:10.1007/s13668-024-00583-3)
Supplement: Supplementary file 1 — Supplementary file1 (DOCX 24 KB) [file 13668_2024_583_MOESM1_ESM.docx]

**Online Resource 1**

**Title:** Miracle fruit, a potential taste-modifier to improve food preferences: A review.

**Journal name:** Current Nutrition Reports

**Authors:** Shashya Diyapaththugama^a^, Getahun Fentaw Mulaw^a^, Madiha Ajaz^a^, Natalie Colson^a^, Indu Singh^a^, Rati Jani^b^

^a^School of Pharmacy and Medical Sciences, Griffith University, Gold Coast, QLD 4222, Australia.

^b^School of Health Sciences and Social Work, Griffith Health, Griffith University. Gold Coast. QLD 4222, Australia.

Corresponding author: Shashya Diyapaththugama

Email address: [shashya.diyapaththugamavidanalage@griffithuni.edu.au](mailto:shashya.diyapaththugamavidanalage@griffithuni.edu.au)

**Table 1.** Nutrient and phytochemical profile of the miracle fruit pulp.

| Nutrient | Content  (Mean or Mean±SD) | Units |
| --- | --- | --- |
| Moisture | 16.44±0.28[1] | % dry weight |
| Proteins | 15.33±4.51[1] | % dry weight |
| Fats | 1.83±0.48[1] | % dry weight |
| Carbohydrates | 65.07±3.95[1] | % dry weight |
| Ashes | 1.33±0.02[1] | % dry weight |
| Dietary fibre | 1.16±0.01[1] | % dry weight |
| Miraculin | 0.29[2] | mg/g of juice of miracle fruit pulp |
| Sodium | 646.67±55.08[1] | mg/100g dry weight |
| Potassium | 1463.33±306.00[1] | mg/100g dry weight |
| Calcium | 423.33±17.90[1] | mg/100g dry weight |
| Phosphorous | 120.96±4.61[1] | mg/100g dry weight |
| Iron | 54.33±14.01[1] | mg/100g dry weight |
| Magnesium | 293.33±25.17[1] | mg/100g dry weight |
| α-tocopherol | 5.8±0.3[3] | mg/100g fresh weight |
| α-tocotrienol | 0.6±0.1[3] | mg/100g fresh weight |
| γ-tocopherol | 1.0±0.1[3] | mg/100g fresh weight |
| Total flavonoids | 9.9±0.5[3] | mg of quercetin equivalents/  100g fresh weight |
| Anthocyanins | 13.5[3] | mg/100g fresh weight |
| Total phenolic content  (Folin- Ciocalteu method) | 1448.3[3] | mg Gallic acid equivalents/  100g fresh weight |
| Epicatechin | 17.8±0.3[3] | mg/100g fresh weight |
| Gallic acid | 10.7±0.2[3] | mg/100g fresh weight |
| Ferulic acid | 5.8±0.1[3] | mg/100g fresh weight |
| Syringic acid | 3.3±0.2[3] | mg/100g fresh weight |
| Rutin | 2.8±0.1[3] | mg/100g fresh weight |
| Quercetin | 1.1±0.1[3] | mg/100g fresh weight |
| Lutein (Carotenoid) | 0.4±0.00[3] | mg/100g fresh weight |
| Ascorbic acid | 28.9±0.9[3] | mg/100g fresh weight |

References:

1. Agblekpe AK, Osseyi E, Dossou J. Potential nutritional values of skin, pulp and seed of miracle fruit (Synsepalum dulcifium). Am J Innov Res Appl Sci. 2016;4:1-7.

2. Demesyeux L, Brym M, Valdes D, Collazo C, Chambers AH. Yield and miraculin content of nine miracle fruit (Synsepalum Dulcificum) morphotypes. Euphytica. 2020;216(11).

3. Du L, Shen Y, Zhang X, Prinyawiwatkul W, Xu Z. Antioxidant-rich phytochemicals in miracle berry (Synsepalum dulcificum) and antioxidant activity of its extracts. Food Chem. 2014;153:279-84.
