# Supplementary material for: Miracle Fruit, a Potential Taste-modifier to Improve Food Preferences: A Review
Source: Curr Nutr Rep. 2024 Oct 3;13(4):867–83. doi: 10.1007/s13668-024-00583-3 (PMC11489218; doi:10.1007/s13668-024-00583-3)
Supplement: Supplementary file 2 — Supplementary file2 (DOCX 23 KB) [file 13668_2024_583_MOESM2_ESM.docx]

**Online Resource 2**

**Title:** Miracle fruit, a potential taste-modifier to improve food preferences: A review.

**Journal name:** Current Nutrition Reports

**Authors:** Shashya Diyapaththugama^a^, Getahun Fentaw Mulaw^a^, Madiha Ajaz^a^, Natalie Colson^a^, Indu Singh^a^, Rati Jani^b^

^a^School of Pharmacy and Medical Sciences, Griffith University, Gold Coast, QLD 4222, Australia.

^b^School of Health Sciences and Social Work, Griffith Health, Griffith University. Gold Coast. QLD 4222, Australia.

Corresponding author: Shashya Diyapaththugama

Email address: [shashya.diyapaththugamavidanalage@griffithuni.edu.au](mailto:shashya.diyapaththugamavidanalage@griffithuni.edu.au)

**Table 2.** Summary of existing reviews on health applications of miracle fruit

| Existing review on health applications  Author (year) | Health applications | | Details of taste modification studies mentioned in the review article*  Author (year) |
| --- | --- | --- | --- |
|  | Health effects | Mechanism |  |
| Ee et al. (2022) | Antioxidant, anti-diabetic, anti-cancer, anti- hyperuricemia, anti-hyperlipidemic, and anti-convulsant effects. | Skin, fruit pulp, leaves, seeds contain phytochemicals such as phenols, flavonoids, tannins, ascorbic acid, epicatechins, gallic acid, etc. which provide these health benefits | Mentioned that Wilken and Satiroff (2012) conducted a pilot study to test the taste alteration of miracle fruit on chemotherapy patients. |
| Akinmoladun (2016) | Antioxidant, anti-diabetic, anti-cancer, anti- hyperuricemia, anti-hyperlipidemic, and anti-convulsant effects. | Phytochemicals in miracle fruit such as alkaloids, lignins, phytosterols, terpenoids, phenolic acids, flavonoids, and amino acids are responsible for health benefits. | Mentioned that Bartoshuk et al. (1974) and Rodrigues et al. (2016)  identified the taste modifying character of miracle fruit on acids which last for 30 minutes. |
| Gómez de Cedrón et al. (2020) | Antioxidant, anti-cancer | Terpenoids, phenolic compounds,  and flavonoids, provide antioxidant activity. Phytochemicals manage oxidative stress to control cancer. | Mentioned that, Wilken and Satiroff (2012) studied miracle fruit to avoid dysgeusia in chemotherapy patients; Wong and Kern (2011)  formed low-calorie diets with miracle fruit to reduce calorie intake; Choi and Garza (2020) studied the taste modification effects of miracle fruit products on different food; Rodrigues et al. (2016) added miracle fruit to sweeten lemonade. |
| Swamy et al. (2014) | Antioxidant, anti-diabetic, anti-cancer | Radical scavenging and antioxidant activity by dihydro-feruloyl-5-methoxytyramine, syringaresinol, coumaric acid, syringic acid etc. | Mentioned that Kurihara and Beidler (1969) purified miraculin; Bartoshuk et al. (1974) described the taste-modifying activity of miracle fruit using HCl and citric acids; Wong and Kern (2011) sweetened a dessert using miracle fruit; Wilken and Satiroff (2012) used miracle fruit to improve the palatability of food in chemotherapy patients. |
| Existing review on health applications  Author (year) | Health applications | | Details of taste modification studies mentioned in the review article*  Author (year) |
|  | Health effects | Mechanism |  |
| Sadhana & Suresh (2020) | Antioxidant, anti-diabetic, anti-hyperuricemia, anti-convulsant | Skin, seeds, pulp, leaf extracts contain phytochemicals including flavonoids which provide health effects. | Mentioned that Wilken and Satiroff (2012) used miracle fruit to change the metallic taste in mouth of chemotherapy patients. |
| Mangla and Kohli (2018) | Anti-diabetic, anti-hyperuricemia, anti-  hyperlipidemic | Phytochemicals in miracle fruit such as anthocyanin, flavanol pigments in fruits and leaves, rutin, gallic acid, ferulic acid, quercetin, epicatechin, myricetin etc. are responsible for health benefits. | Cited Bartoshuk et al. (1974) in mentioning that miracle fruit provides taste modification. |

*According to the summary, none of the existing reviews on miracle fruit comprehensively synthesized all available original research evidence on taste modification.

References

Akinmoladun, A. C. (2016). Effect of Synsepalum dulcificum berry extract on oxidative stress and hepatotoxicity indices, following subacute administration in normal rats. *FUTA J. Res. Sci*, *12*, 167-177.

Bartoshuk, L. M., Gentile, R. L., Moskowitz, H. R., & Meiselman, H. L. (1974). Sweet taste induced by miracle fruit (Synsepalum dulcificum). *Physiology & Behavior*, *12*(3), 449-456.

Choi, S. E., & Garza, J. (2020). Effects of different miracle fruit products on the sensory characteristics of different types of sour foods by descriptive analysis [Article]. *Journal of Food Science*, *85*(1), 36-49. https://doi.org/10.1111/1750-3841.14988

Ee, J. W., Velaga, A., Guad, R. M., Subramaniyan, V., Fuloria, N. K., Choy, K. W., Fuloria, S., & Wu, Y. S. (2022). Deciphering Synsepalum dulcificum as an Arising Phytotherapy Agent: Background, Phytochemical and Pharmacological Properties with Associated Molecular Mechanisms. *Sains Malaysiana*, *51*(1), 199-208.

Gómez de Cedrón, M., Wagner, S., Reguero, M., Menéndez-Rey, A., & Ramírez de Molina, A. (2020). Miracle berry as a potential supplement in the control of metabolic risk factors in cancer. *Antioxidants*, *9*(12), 1282.

Kurihara, K., & Beidler, L. M. (1969). Mechanism of the action of taste-modifying protein [Letter]. *Nature*, *222*(5199), 1176-1179. https://doi.org/10.1038/2221176a0

Lipatova, O., & Campolattaro, M. M. (2016). The miracle fruit: an undergraduate laboratory exercise in taste sensation and perception. *Journal of Undergraduate Neuroscience Education*, *15*(1), A56. https://www.ncbi.nlm.nih.gov/pmc/articles/PMC5105965/pdf/june-15-56.pdf

Mangla, B., & Kohli, K. (2018). Pharmaceutical and Therapeutic Potential of Miraculin and Miracle Berry: doi. org/10.26538/tjnpr/v2i1. 3. *Tropical Journal of Natural Product Research (TJNPR)*, *2*(1), 12-17.

Rodrigues, J. F., Andrade, R. D. S., Bastos, S. C., Coelho, S. B., & Pinheiro, A. C. M. (2016). Miracle fruit: An alternative sugar substitute in sour beverages [Article]. *Appetite*, *107*, 645-653. https://doi.org/10.1016/j.appet.2016.09.014

Sadhana H M, S. J., Hamsalakshmi. (2020). Synsepalum Dulcificum: A Review. International Journal of Research in Pharmaceutical Sciences, 4208-4213.

Swamy, K. B., Hadi, S. A., Sekaran, M., & Pichika, M. R. (2014). The clinical effects of Synsepalum dulcificum: a review [Review]. *Journal of Medicinal Food*, *17*(11), 1165-1169. https://doi.org/10.1089/jmf.2013.3084

Wilken, M. K., & Satiroff, B. A. (2012). Pilot study of "miracle fruit" to improve food palatability for patients receiving chemotherapy [Article]. *Clinical Journal of Oncology Nursing*, *16*(5), E173-E177. https://doi.org/10.1188/12.CJON.E173-E177

Wong, J. M., & Kern, M. (2011). Miracle fruit improves sweetness of a low-calorie dessert without promoting subsequent energy compensation [Article]. *Appetite*, *56*(1), 163-166. https://doi.org/10.1016/j.appet.2010.10.005
