# Supplementary material for: Miracle Fruit, a Potential Taste-modifier to Improve Food Preferences: A Review
Source: Curr Nutr Rep. 2024 Oct 3;13(4):867–83. doi: 10.1007/s13668-024-00583-3 (PMC11489218; doi:10.1007/s13668-024-00583-3)
Supplement: Supplementary file 3 — Supplementary file3 (DOCX 16 KB) [file 13668_2024_583_MOESM3_ESM.docx]

**Online Resource 3**

**Title:** Miracle fruit, a potential taste-modifier to improve food preferences: A review.

**Journal name:** Current Nutrition Reports

**Authors:** Shashya Diyapaththugama^a^, Getahun Fentaw Mulaw^a^, Madiha Ajaz^a^, Natalie Colson^a^, Indu Singh^a^, Rati Jani^b^

^a^School of Pharmacy and Medical Sciences, Griffith University, Gold Coast, QLD 4222, Australia.

^b^School of Health Sciences and Social Work, Griffith Health, Griffith University. Gold Coast. QLD 4222, Australia.

Corresponding author: Shashya Diyapaththugama

Email address: [shashya.diyapaththugamavidanalage@griffithuni.edu.au](mailto:shashya.diyapaththugamavidanalage@griffithuni.edu.au)

**Table 3.** PICO framework defined research question for the review

| Population | Human subjects |
| --- | --- |
| Intervention | Any study design examining the association between miracle fruit/ miraculin and taste perceptions and food preferences. |
| Comparison | Taste perceptions and food preferences before miracle fruit/ miraculin administration or after placebo administration. |
| Outcomes | Perceived taste quality, taste intensity, food liking, food preferences, dietary intake, diet quality |

**Table 4.** Search strategy

| **Search strategy based on PICO framework** | |
| --- | --- |
| 1 | human* |
| 2 | (adult*male* or female*) |
| 3 | 1 or 2 |
| 4 | exp SYNSEPALUM/ or exp MIRACULIN PROTEIN/ or exp SYNSEPALUM DULCIFICUM/ |
| 5 | (miracle berr* or miracle fruit* or miraculin) |
| 6 | (Richadella or Richadella dulcific*) |
| 7 | 4 or 5 or 6 |
| 7 | (taste or taste perception or taste quality or taste modif* or taste alter* or taste sensitivit*) |
| 9 | (food preference* or food liking or food intake) |
| 10 | (diet or diet quality) |
| 11 | (sour* or sweet* or salt* or bitter*or tart* or acid*) |
| 12 | (food or food product or fruit* or vegetable* or dessert or beverage) |
| 13 | 7 or 8 or 9 or 10 or 11 or 12 |
| 14 | 3 and 7 and 13 |
| **The syntax used in this search strategy, Boolean operators (AND, OR and NOT), truncations (e.g., sour*) were adjusted where necessary according to the requirements of each database.* | |
