# Supplementary material for: Miracle Fruit, a Potential Taste-modifier to Improve Food Preferences: A Review
Source: Curr Nutr Rep. 2024 Oct 3;13(4):867–83. doi: 10.1007/s13668-024-00583-3 (PMC11489218; doi:10.1007/s13668-024-00583-3)
Supplement: Supplementary file 4 — Supplementary file4 (DOCX 53 KB) [file 13668_2024_583_MOESM4_ESM.docx]

**Online Resource 4**

**Title:** Miracle fruit, a potential taste-modifier to improve food preferences: A review.

**Journal name:** Current Nutrition Reports

**Authors:** Shashya Diyapaththugama^a^, Getahun Fentaw Mulaw^a^, Madiha Ajaz^a^, Natalie Colson^a^, Indu Singh^a^, Rati Jani^b^

^a^School of Pharmacy and Medical Sciences, Griffith University, Gold Coast, QLD 4222, Australia.

^b^School of Health Sciences and Social Work, Griffith Health, Griffith University. Gold Coast. QLD 4222, Australia.

Corresponding author: Shashya Diyapaththugama

Email address: [shashya.diyapaththugamavidanalage@griffithuni.edu.au](mailto:shashya.diyapaththugamavidanalage@griffithuni.edu.au)

Studies from databases/registers **(n = 436)**

Scopus (n = 167)

Web of Science (n = 164)

MEDLINE (n = 80)

CINAHL (n = 23)

Citation searching (n = 2)

**Identification**

Studies included in review **(n = 16)**

Studies excluded **(n = 207)**

Irrelevant title and/or abstract (n=207)

Studies not retrieved **(n = 0)**

Studies assessed for eligibility **(n = 59)**

Studies sought for retrieval **(n = 59)**

Studies screened **(n = 266)**

Studies excluded **(n = 43)**

Protocol paper (n = 1)

Wrong outcomes (n = 3)

Review article (n = 21)

Wrong indication (n = 2)

Wrong intervention (n = 10)

Full text unavailable (n = 6)

References removed **(n = 170)**

Duplicates identified manually (n = 13)

Duplicates identified by Covidence (n = 157)

Marked as ineligible by automation tools (n = 0)

Other reasons (n = 0)

**Screening**

**Included**

**Figure 1.** PRISMA flowchart of study identification, screening, and inclusion process [1]

Reference

1. Page MJ, McKenzie JE, Bossuyt PM, Boutron I, Hoffmann TC, Mulrow CD, et al. The PRISMA 2020 statement: an updated guideline for reporting systematic reviews. Bmj. 2021;372.
