# Supplementary material for: Miracle Fruit, a Potential Taste-modifier to Improve Food Preferences: A Review
Source: Curr Nutr Rep. 2024 Oct 3;13(4):867–83. doi: 10.1007/s13668-024-00583-3 (PMC11489218; doi:10.1007/s13668-024-00583-3)
Supplement: Supplementary file 5 — Supplementary file5 (DOCX 28 KB) [file 13668_2024_583_MOESM5_ESM.docx]

**Online Resource 5**

**Title:** Miracle fruit, a potential taste-modifier to improve food preferences: A review.

**Journal name:** Current Nutrition Reports

**Authors:** Shashya Diyapaththugama^a^, Getahun Fentaw Mulaw^a^, Madiha Ajaz^a^, Natalie Colson^a^, Indu Singh^a^, Rati Jani^b^

^a^School of Pharmacy and Medical Sciences, Griffith University, Gold Coast, QLD 4222, Australia.

^b^School of Health Sciences and Social Work, Griffith Health, Griffith University. Gold Coast. QLD 4222, Australia.

Corresponding author: Shashya Diyapaththugama

Email address: [shashya.diyapaththugamavidanalage@griffithuni.edu.au](mailto:shashya.diyapaththugamavidanalage@griffithuni.edu.au)

**Figure 2.** Flow chart of studies organised by study design.
